# Does Case Management Provide Support for Staff Facing Frequent Users of Emergency Departments? A Comparative Mixed-Method Evaluation of ED Staff Perception

**DOI:** 10.1186/s12873-021-00481-9

**Published:** 2021-08-04

**Authors:** Michael von Allmen, Véronique S. Grazioli, Miriam Kasztura, Oriane Chastonay, Joanna C. Moullin, Olivier Hugli, Jean-Bernard Daeppen, Patrick Bodenmann

**Affiliations:** 1 Department of Vulnerabilities and Social Medicine, University Center for General Medicine and Public Health, Lausanne, Switzerland; 2 Faculty of Health Sciences, Curtin University, Perth, Australia; 3 Emergency Department, University Hospital, Lausanne, Switzerland; 4 Addiction Medicine, Department of Psychiatry, Lausanne University Hospital, University of Lausanne, Lausanne, Switzerland

**Keywords:** Frequent users of emergency departments, Case management, Emergency service, Staff

## Abstract

**Objective:**

Frequent users of emergency departments (FUED) account for a disproportionate number of emergency department (ED) visits and contribute to a wide range of challenges for ED staff. While several research has documented that case management (CM) tailored to FUED leads to a reduction in ED visits and a better quality of life (QoL) among FUED, whether there is added value for ED staff remains to be explored. This study aimed to compare, among staff in two academic EDs in Switzerland (one with and one without CM), the FUED-related knowledge, perceptions of the extent of the FUED issue, FUED-related work challenges and FUEDs’ legitimacy to use ED.

**Method:**

Mixed methods were employed. First, ED physicians and nurses (*N* = 253) of the two EDs completed an online survey assessing their knowledge and perceptions of FUEDs. Results between healthcare providers working in an ED with CM to those working in an ED without CM were compared using independent two-sided T-tests. Next, a sample of participants (*n* = 16) took part in a qualitative assessment via one-to-one interviews (*n* = 6) or focus groups (*n* = 10).

**Results:**

Both quantitative and qualitative results documented that the FUED-related knowledge, the extent FUED were perceived as an issue and perceived FUEDs’ legitimacy to use ED were not different between groups. The level of perceived FUED-related challenges was also similar between groups. Quantitative results showed that nurses with CM experienced more challenges related to FUED. Qualitative exploration revealed that lack of psychiatric staff within the emergency team and lack of communication between ED staff and CM team were some of the explanations behind these counterintuitive findings.

**Conclusion:**

Despite promising results on FUEDs’ QoL and frequency of ED visits, these preliminary findings suggest that CM may provide limited support to ED staff in its current form. Given the high burden of FUED-related challenges encountered by ED staff, improved communication and FUED-related knowledge transfer between ED staff and the CM team should be prioritized to increase the value of a FUED CM intervention for ED staff.

**Supplementary Information:**

The online version contains supplementary material available at 10.1186/s12873-021-00481-9.

## Background

Frequent users of emergency departments (FUED) have been the focus of increasing attention over the past decade. The term FUED refers to people who visit the emergency department (ED) 5 or more times in a 12-month-period. They account for 4 to 16% of total ED users and 12 to 47% of ED visits, contributing to ED overcrowding and increasing health care costs [1–3].

FUED are a heterogenous group of patients sharing common characteristics [4]. Compared to ED patients who do not fulfil the FUED criteria, FUED have a higher prevalence of somatic and psychiatric comorbidities, psychological conditions, addiction [1, 5] and social issues [1, 6]. They often cumulate vulnerabilities [7] leading to a higher mortality rate [8], and a poorer quality of life (QoL) [9]. Furthermore, FUED are likely to report feelings of discrimination, increasing their risk of being in situation of vulnerability [10].

In response, significant research efforts have been dedicated to develop interventions tailored to FUED, such as case management (CM) [11]. CM oriented to FUED is a process conducted by health professionals (i.e., nurse, physician, social workers) inside and outside the ED, once any urgent issues have been solved. It aims to empower patients and increase their ability to interact with the healthcare system [12]. Published literature indicates that CM generally leads to a reduction in ED visits and healthcare costs [2, 11–13]. Besides, it also improves FUEDs’ QoL [9].

Surprisingly, there is very limited exploration regarding ED staff experiences caring for FUED. We are aware of only two qualitative studies involving ED staff on this topic, conducted in the USA and Singapore [14, 15]. Both studies report that staff faced challenges in addressing FUEDs’ needs and experienced feelings of fatigue, failure and reduced mood. ED staff have a high prevalence of burnout [16], and any potential cause needs to be investigated. CM may alleviate these challenges. To our knowledge however, no study has explored whether there is added value for ED staff caring for FUED.

Therefore, this study was designed to address this gap in the literature. It aims at comparing FUED-related knowledge, the perception of the extent of FUED issue, perceived work challenges related to FUED and the perceived legitimacy of FUED ED visits between ED staff working in two academic ED only 45 miles apart, one with a nine-years’ experience of CM implemented and one without it.

This study was nested in a larger ongoing research project that aimed to develop and implement a CM intervention tailored to FUEDs in the public hospitals with ED in the French-speaking region of Switzerland (project number 2018–00442) [17].

## Method

### ED hospitals

Research was conducted in two Swiss university hospitals (45,000 [with CM] and 75,000 [without CM] annual consultations). CM is an on-demand intervention provided by an external consultation team once a FUED is identified by the ED team. Also of note, the ED without CM has an integrated psychiatric emergency unit, whilst this is an external consultation service in the ED with CM.

### Quantitative methods

#### Sample

Participants (*N* = 253) were ED staff working in these two Swiss university hospitals, divided by staff with CM (*n* = 100) and staff without CM (*n* = 153).

#### Measures

A 12-item online survey was developed to measure variables related to the FUED issue, summarized below and presented in Appendix 1. The survey was based on a version developed by a panel of experts involved with FUED and used in ongoing research [18, 19]. As described in Chastonay et al. [19], the panel conducted a series of sessions to develop a set of items exploring ED staff’s perceptions regarding FUED and associated issues. The survey was tested by ED staff (*Lausanne university hospital, CHUV*; *N* = 14). The version used in this study was composed by a selection of original items matching with its variables [18, 19].

##### Demographic variables

The online survey included demographic variables (i.e., age, sex, years of practical experience and profession).

##### Dependent variables

Participants were asked to indicate the extent to which they agreed with statements related to FUED. First, a statement explored the participants’ own perception of their FUED-related knowledge. Next, their actual FUED- related knowledge was explored by assessment of their knowledge of FUEDs’ attributes as reported in literature [1, 5, 6]. Then, statements explored the extent FUED are perceived as an issue, perceived level of FUED ED visit, perceived legitimacy of FUED ED visits and perception of FUED-related challenges (i.e., feeling of burnout, feeling of helplessness, organizational issues and FUED characteristics). (See Table 1).

**Table 1:** Dependent variables

Dependent variable	Measurement^c^
FUED-related knowledge	
*level of perceived knowledge*	4-point Likert-type scale statement
*actual knowledge score*	Agreement mean score of fifteen 10-point Likert-type scale statements describing FUED characteristics supported by existing evidence [17] (see appendix question 11)
Extent FUED are perceived as an issue	4-point Likert-type scale statement
Perceived level of FUED ED visit	4-point Likert-type scale statement
Perceived legitimacy of FUED ED visits FUED	10-pointl Likert-type scale statement
FUED-related work challenges:	Sixteen 10-point Likert-type scale statements based on known FUED-related work challenges [18] (see appendix question 8) summarize in 4 dependent variables after a principal component analysis (see data management)
-Feeling of burnout	
-Feeling of helplessness	
-Organizational issues	
-FUED characteristics	

##### Independent variables

Type of emergency care (with/ without CM) served as the independent variable (hereafter referred as groups), whereas sex (male/female), profession (nurse/ physician) and years of practice (0–6 years /> 6 years, 6 years being the median) were used to stratify the analysis (hereafter referred as subgroups).

#### Procedures

From July 2018 to September 2018, all ED nurses and physicians of both hospitals were invited to complete the online survey. Email reminders were sent until at least a 60% [20] participation rate was achieved in both groups. All procedures were approved by the Swiss Ethics Committee (project number 2018–00442) [17].

#### Analyses

First, two-sided independent samples t-test were conducted with SPSS 25 to compare perceptions of FUED (i.e., dependent variables) between the groups with or without CM. Then, stratification was conducted by further t-tests in subgroups (i.e., independent variables). The significance level was set at *p = .05.*

#### FUED-related challenges

The 16 variables regarding FUED-related challenges were subject to a principal component analysis (PCA). Suitability of data for factorial analysis was supported by correlation matrix inspection revealing coefficients of 0.3 and above, value of Kasier-Meyer-Olkin (0.87) and statistical significance of Bartlett’s Test of Sphericity. PCA revealed the presence of four components with eigenvalues exceeding one, explaining 37.1, 10.2, 9.2, 6.8% of the variance. Accordingly, a four-component solution consistent to literature [1, 5, 6] was selected (hereafter referred as feeling of helplessness, organizational issues, FUED characteristics and feeling of burnout).

### Qualitative methods

#### Sample

ED Nurses and physicians of both hospitals have received an email invitation to participate. Among them, 16 professionals were showed interest in participate and were included (with (*n* = 6) and without (*n* = 10) CM).

#### Measures

A grid of open-ended questions (see Appendix 2) was developed and employed in semi-structured interviews and focus groups to explore the perceived level of knowledge regarding FUED, extent FUED are perceived as an issue, perceived level FUED visit the ED, perceived legitimacy of FUED ED visits and perception of FUED-related challenges.

#### Procedures

Qualitative exploration regarding nurses was done through two focus groups (60 min each) by two study authors (MvA, VG), one with nurses working in the ED with CM (*n* = 3*)* and the other with those in the ED without CM *(n* = 7*)*. Qualitative assessment for physicians from EDs with CM (*n* = 3) and without CM (*n* = 3) was done through semi-structured interviews (20–45 min, conducted by MvA), since focus groups were not possible due to physicians’s agenda contraints. Conversations were recorded after receiving participants’ informed consent.

#### Data management and analysis plan

Interviews records were transcribed *verbatim*. Conventional content analysis was conducted on Atlas. Ti version 7 [21]. Initial coding was conducted by study authors (MvA, VG) using a line-by-line technique, whereby coders narrated the actions occurring in the interviews [22, 23]. Following independent initial coding, a codebook was created in consensus meetings, pooling codes and eliminating idiosyncratic or redundant ones. Next, we used the codebook to independently double-code 10% of the interviews until adequate intercoder consistency (80%) was attained [22, 23]. Once adequate intercoder consistency was established, the remaining interviews were coded independently by MvA.

## Results

### Quantitative results

In total, 296 participants completed the survey (60% in the total staff of both hospitals). Of those, 85.5% completed more than demographic questions in the survey (i.e., information regarding age, sex, years of practical experience and profession), resulting in a final sample of 253 participants. Table 2 presents demographics by groups (CM, no-CM). Table 3 (c.f additional materials) presents descriptive statistics and t-tests results by groups (CM, no-CM) and within subgroups (physicians, nurses, males, females, 1–6 years of experience, > 6 years of experience).

**Table 2:** Demographics results

	CM		No-CM		*X*^*2*^
	*S*	*%*	*S*	*%*	
Gender					.070
Female	61	61	110	71.9	
Male	39	39	43	28.1	
Professions					.779
Physician	31	31	50	32.7	
Nurses	69	69	103	67.3	
	CM	No-CM	Statistics		*P*-value
Years of practical experience	7.14	9.83	t(250) = −2.741		.007

**Table 3:** Results of t-tests and descriptive statistics by hospital (CM/ no CM) and within subgroups

Variable	All		Physicians	Nurses			Male		Female		1–6 y. of exp.		> 6 y. of exp.	
	CM		No CM	CM			No CM		CM		No CM		CM	
1. Level of perceived knowledge														
M (SD)	2.63 (.726)	2.83 (.65)	2.62 (.82)	2.81 (.68)	2.63 (.689)	2.84 (.641)	2.43 (.765)	2.73 (.679)	2.75 (.68)	2.87 (.64)	2.72 (.64)	2.97 (.605)	2.49 (.82)	2.74 (.67)
n	142	97	47	29	68	95	40	37	60	102	58	61	39	80
95% CI	.021–.383		−.154–.534		−.001–.420		−.035–.620		−.088–.333		.016–.470		−.053–.553	
t	2.2*		1.08		1.97		1.77		1.15		2.12*		1.65	
2. Actual knowledge score														
M (SD)	6.50 (1.39)	6.61 (1.51)	6.67 (1.18)	6.87 (1.29)	6.58 (1.48)	6.47 (1.6)	6.67 (1.37)	6.38 (1.23)	6.56 (1.4)	6.69 (1.61)	6.47 (1.41)	6.47 (1.54)	6.8 (1.35)	6.7 (1.46)
n	96	142	29	47	67	95	37	40	60	102	58	61	39	80
95% CI	−.378–.385		−.377–.799		−.592–.387		−.877–.310		−.365–.629		−.530–.545		−.700–.412	
t	.018		.714		−.415		.952		(159) = .525		.026		−.513	
3. Extent FUED are perceived as an issue														
M (SD)	2.49 (.703)	2.57 (.572)	2.19 (.703)	2.54 (.544)	2.62 (.66)	2.58 (.586	2.46 (.79)	2.53 (.550)	2.51 (.649)	2.58 (.582)	2.46 (.721)	2.57 (.558)	2.54 (.682)	2.58 (.585)
n	100	151	31	48	69	103	39	43	61	108	61	65	39	85
95% CI	−.087–.246		.068–.629		−.231–.150		−.229–.376		−.117–.267		−.118–.339		−.198–.274	
t	.943		2.47*		−.422		.483		.773		.956		.219	
4. Perceived level of FUED ED visit														
M (SD)	2.18 (.757)	2.17 (.725)	2.16 (.638)	2.29 (.771)	2.19 (.809)	2.11 (.699)	2.08 (664)	2.40 (.849)	2.25 (.809)	2.07 (.651)	2.25 (.789)	2.32 (.773)	2.08 (.703)	2.05 (.671)
n	100	151	31	48	69	103	39	43	61	108	61	65	39	85
95% CI	−.202–.173		−.201–.462		−.310–.147		−.019–.656		−.412–.068		−.198–.353		−.291–.231	
t	−.152		.435		−.704		1.88		− 1.42		.555		.821	
5. Perceived legitimacy of FUED ED visits														
M (SD)	4.22 (2.34)	3.89 (2.23)	4.07 (1.86)	3.70 (2.28)	4.29 (2.53)	3.98 (2.20)	4.16 (2.48)	3.81 (2.45)	4.26 (2.26)	3.92 (2.14)	3.7 (2.03)	3.94 (2.3)	5.05 (2.58)	3.86 (2.18)
n	99	146	30	47	69	99	38	42	61	104	61	63	38	83
95% CI	−.912–.251		−1.335-.626		−1.03-.415		−1.45-.750		− 1.03-.357		−.540–1.003		−2.094- -.300	
t	− 1.12		−.733		−.844		−.632		−.962		.594		−2.64*	
6. Perception of FUED-related challenges: Feeling of burnout^b^														
M (SD)	6.63 (1.83)	6.70 (2.13)	6.32 (1.87)	6.48 (1.74)	2.76 (1.80)	6.80 (2.29)	6.61 (1.76)	6.14 (1.97)	6.63 (1.89)	6.92 (2.16)	6.82 (1.86)	6.63 (1.95)	6.33 (1.78)	6.74 (2.28)
n	100	151	31	48	69	103	39	43	61	108	61	65	39	85
95% CI	−.44–.58		−.67–.98		−.58–.66		−1.29-.35		−.37–.94		−.86–.48		−.41–1.23	
t	.276		.378		.120		−1.15		.863		−.556		1.079	
7. Perception of FUED-related challenges: Feeling of helplessness^b^														
M (SD)	6.63 (2.15)	5.95 (2.35)	5.98 (2.36)	6.37 (2.15)	6.93 (1.99)	5.76 (2.42)	6.34 (2.02)	5.83 (2.50)	6.82 (2.22)	6.01 (2.30)	6.51 (2.23)	6.00 (2.14)	6.82 (2.02)	5.99 (2.44)
n	100	151	31	48	69	103	39	43	61	108	61	65	39	85
95% CI	−1.25- -.09		−.63–1.42		−1.86- -4.72		− 1.52-.49		− 1.53- -0.095		− 1.28 -.26		−1.71-.06	
t	−2.3		.768		−3.3**		−1.01		−2.23*		−1.32		− 1.85	
8. Perception of FUED-related challenges: Organizational issues^b^														
M (SD)	6.6 (2.11)	6.75 (2.14)	6.46 (2.29)	7.37 (2.08)	6.67 (2.04)	6.46 (2.12)	6.81 (1.86)	6.87 (2.32)	6.47 (2.26)	6.70 (2.07)	6.69 (2.01)	6.80 (1.84)	6.47 (2.28)	6.71 (2.37)
n	100	148	31	48	69	100	39	43	61	105	61	65	39	89
95% CI	−.39–.69		−.096–1.89		−.85–.44		−.87–.99		−.45–.91		.12–.344		.232–.456	
t	.538		1.79		−.632		.138		.664		.346		.511	
9. Perception of FUED-related challenges: FUED characteristics^a^														
M (SD)	6.88 (1.45)	6.73 (1.76)	7.06 (1.39)	6.96 (1.54)	6.80 (1.48)	6.61 (1.85)	7.01 (1.30)	6.36 (1.93)	6.80 (1.54)	6.87 (1.67)	6.88 (1.45)	6.84 (1.61)	6.89 (1.46)	6.67 (1.86)
n	100	151	31	48	69	103	39	43	61	108	61	65	39	85
95% CI	−.57–.26		−.78–.58		−.71–.34		−1.38-.08		−.44–.59		−.58–.50		−.88–.45	
t	−.732		−.290		−.692		−1.77		.281		−.141		−.648	

*p < 0.05, **p < 0.01, ***p < 0.001

### Demographic results

Participants were predominately female (67.6%), reflecting the current proportion among health professionals in Switzerland [24]. Of the overall sample, 32% were physicians and 68% were either nurses or nurse assistants. Years of practical experience median was 6 years (*IQR* = 10) and 67.2% were between 30 to 49 years old.

#### Perceived level of knowledge and knowledge score

Overall, the group with CM perceived their knowledge of FUED as significantly better than those working in a ED without CM. In subgroup analyses of ED staff with less work experience, their perception of their FUED knowledge was better in those with CM compare to those without. However, the actual knowledge score regarding FUED characteristics was not significantly different between groups and subgroups.

#### Extent of the FUED issue

Although it was not significantly different between groups, the physician subgroup with CM saw FUED as less of an issue than physicians without CM.

#### Perceived level FUED visit the ED

There was no significant difference in the perceived level of FUEDs’ ED use between groups and subgroups.

#### Perceived legitimacy of FUEDs’ ED visits

Legitimacy was not rated differently between groups. However, the more experienced healthcare provider subgroup in the ED with CM were more prone to consider FUED less legitimate to consult ED compared to those without it.

#### Perception of FUED-related challenges

Whereas perception of most challenges (i.e., feeling of burnout, organizational issues, FUED characteristics and feeling of helplessness) was not significantly different between groups, helplessness scores were significantly higher in nurses with than in those without CM.

### Qualitative results

Participants (*N* = 16) were predominately females (87.5%). Of the overall sample, 37.5% were physicians and 62,5% were nurses. Of physicians, 83% were chief residents and 17% senior physician certified in emergency medicine. Nurses’ years of practical experience median was 9 years (*IQR* = 9). Content analysis identified five main themes. Original quotes in French and translated in English are presented in Appendix 3.

### General knowledge of the FUED population and their characteristics

General knowledge of FUED was considered insufficient among participants with and without CM (e.g., *physician 3, no-CM*: “It is not a population we are informed about. I have heard very little of recurrent ED patients as a specific entity or type of patient that would require specific management”).

### Extent of FUED issue

Both groups reported they frequently encountered FUED (e.g., *physician 3, no-CM*: “It is still important in terms of the number of patients and frequency of emergency room visits”).

### FUED legitimacy to consult ED

FUEDs’ legitimacy to consult ED was considered equally low between participants with and without CM, due to absence of medical conditions justifying ED consultations (e.g, *physician 1, CM*: “the place for these people is not emergency rooms”) (e.g., *physician 2, no-CM*: “It’s people who are in good health (…) don’t have many comorbidities”).

### Challenges encountered in the management of FUED

Participants with and without CM experienced the same range of challenges when providing healthcare to FUED (e.g.*, physician 3, CM*:” The first thing in these patients is: time consuming, annoying and generates negative counter-transfers”; *physician 2, CM*: “We just can’t heal them. So yes, it awakens a feeling of helplessness in the team and fatigue”).

### Perceptions of FUEDs management, its strengths and weaknesses

Participants in both groups saw numerous benefits of CM tailored to FUED, such as “adapting patient care to their needs and demands” or “coordinating FUEDs’ healthcare network”. Negative aspects of CM were predominantly raised in the nurse subgroup with CM. Reported issues were a lack of information and feedback regarding CM activities (*Nurses’ focus group, CM*: “I wasn’t aware that they were actually doing all this (…) we have less information on what the “vulnerable populations” team (i.e., CM team) do (…) We’re potentially biased because it’s suddenly patients we don’t see anymore and we don’t necessarily realize”). Furthermore, negative evaluation of psychiatric management for FUED was also pointed out (*Nurses’ focus group, CM*: “When you see someone who comes in a recurring way (...) and a quarter of an hour after coming down from a psychiatric consultation, you can’t say it’s efficient or well-done care”).

## Discussion and conclusion

This study is the first quantitative and qualitative exploration of the potential perceived added-value of CM for ED staff, by comparing the perceptions of FUED by ED staff with and without a CM service.

Unexpectedly, in both quantitative and qualitative results, FUED-related knowledge was no better in CM group despite a higher subjective appreciation of it from physicians with CM. These findings suggest that CM for FUED does not contribute to a knowledge transfer to ED staff. To enhance this transfer, active learning approaches conducted by the CM team may be used (e.g., workshops or feed-back sessions on specific patients).” [25].

Hudon et al. found in a primary care setting that CM, by reducing the FUEDs’ psychological distress, made caregivers feel more confident in dealing with FUED challenges [26]. In the ED setting, our results did not come to a similar conclusion. Paradoxically, quantitative results revealed a higher level of helplessness in nurses with CM. The hypothesis is that these results may pertain to confounding factors. First, profession discrepancy might be explained by the confounding effect of professional status (e.g., level of self-awareness and expectations of oneself, difficulty to admit lack of competency) [27]. Furthermore, management of psychiatric emergencies were quite different between the two EDs and may have confounded CM perception. The external psychiatrist consultation service in the ED with CM was subject to negative evaluation from nurses in qualitative exploration. An integrated psychiatric unit may provide greater support to staff facing FUED psychiatric and behavioral issues, representing an important part of FUED care [17]. In addition, qualitative exploration revealed that CM activity was considered not visible enough. Specifically, participants highlighted a lack of feedback from the CM team concerning referred FUED. The insufficient communication between CM and ED teams prevented ED staff from being informed of the CM team’s successes and failures. A better communication between ED and CM team may help address ED staff’s feeling of helplessness.

CM has been proven to reduce FUED consultations in ED [17]. However, the perceived level FUED visit the ED was not quantitatively different between groups. That said, physicians with CM tended to perceive FUED as less of an issue compared to those without. This may also pertain to the fact that nurses and physician are not exposed to FUED-challenges in the same way.

Perception of FUEDs’ legitimacy to use ED did not appear to be impacted by CM implementation. Surprisingly, the qualitative analysis revealed that participants in both groups considered FUEDs’ ED visits as inappropriate. This does not match reality, as most FUEDs’ visits are triggered by objective acute healthcare needs [1, 28]. .Studies conducted in psychiatry demonstrate that staff knowledge is an important factor to foster empathy towards a stigmatized population [29]. The general lack of FUED-related knowledge may explain why both groups perceived FUED to lack legitimacy. Increasing the knowledge transfer through CM team might also address FUEDs’ perception of discrimination.

This study has several limitations. First, the quantitative survey was not previously validated beyond face validity, although it was used in a previous studies [18, 19]. That being said, the survey development went through an expert committee and iterative testing. Second, the study design did not allow for the control of confounding factors. However, triangulation of quantitative and qualitative data strengthened the validity of the analysis. Generalizability of data is also increased by the EDs studied (i.e., two out of five university EDs in Switzerland).

Although preliminary, our findings suggest two recommendations for allowing CM to address FUED challenges experienced by ED staff. First, good communication between ED staff and the CM team is important to support ED staff in their challenges to care for FUED; it contributes to knowledge transfer and eventually decrease perception of FUED illegitimacy to visit ED. Second, we recommend reinforcing collaboration between ED staff and psychiatrists to help address FUED care complexity, by adding a psychiatrist to the CM team if no psychiatry team is present in the ED.

To conclude, despite promising results on FUEDs’ QoL and ED visits, CM may provide limited support to ED staff in its current form. Given the high burden of FUED-related challenges encountered by ED staff, improved communication and FUED-related knowledge transfer between ED staff and the CM team should be prioritized to increase the CM added-value for ED staff.

## Supplementary Information

**Additional file 1.**

## Data Availability

The datasets used and/or analyzed during the current study are available from the corresponding author on reasonable request.

## Abbreviations

FUED: Frequent users of emergency department
ED: Emergency department
QoL: Quality of life
CM: Case management
PCA: principal component analyses
